# Author and Journal Self-Citation in General Surgery Original Research Articles

**DOI:** 10.7759/cureus.88858

**Published:** 2025-07-27

**Authors:** Dhairya Shah, Lucas Gerbasi, Megan A Flores, Tanja Gunsberger, John Ashurst

**Affiliations:** 1 Arizona College of Osteopathic Medicine, Midwestern University, Glendale, USA; 2 Research, Midwestern University, Glendale, USA; 3 Surgery and Anesthesia, Midwestern University, Glendale, USA; 4 Emergency Medicine, Kingman Regional Medical Center, Kingman, USA

**Keywords:** authorship, bibliometric analyses, country self-citation, scientific publishing, self-citation rate

## Abstract

Background: Citations are an indicator of an article’s visibility, significance, impact, and attention in journals. Evidence has suggested that there may be increasing questionable citation practices by authors to achieve “scores and win rewards." It poses the question of whether high citation counts are a realistic metric for an individual’s mark of influence or an attempt at the misrepresentation of performance to game citation scores.

Objective: This study aimed to investigate the difference in author and journal self-citation rates in three high-impact general surgery journals.

Methods: A retrospective cohort of all original research articles published from January 1, 2022, through December 31, 2022, in the Journal of the American Medical Association Surgery (JAMA Surg, Impact Factor (IF) 16.7), Annals of Surgery (AOS, IF 13.8), and the Journal of the American College of Surgeons (ACS, IF 6.5) were reviewed. Data was collected on the total number of authors, references, author self-citations, journal self-citations, and the country associated with the last author. The Kruskal-Wallis test was used to compare continuous data between journals, and all correlations were calculated using Spearman correlation coefficients. An average self-citation index was calculated for every country represented in each journal and plotted on an area map.

Results: A total of 558 articles, 6,399 authors, 19,943 references, 32 states in the U.S., and 25 countries were reviewed and included in the final analysis. Author self-citations accounted for 28.4% (N = 1,819) of all references studied, with a statistically significant difference between JAMA Surg and AOS vs. ACS, and no differences between JAMA Surg and AOS (JAMA Surg 3 and AOS 3 vs. ACS 2; p < 0.001), with data represented as group medians. Authors self-cited themselves a total of 3,852 times, with first authors accounting for 17.1% (N = 658) and last authors accounting for 19.6% (N = 755) of all self-citations. A significant positive correlation was noted between the total number of authors and the number of authors self-citing (r(556) = 0.386; p < 0.001). A significant, strong positive correlation was also noted between the total times authors self-cited and the total times the first and last authors self-cited (first author: r(556) = 0.582, p < 0.001); last author: r(556) = 0.634, p < 0.001). Overall, journal self-citations accounted for 6.7% (N = 1337) of all cited manuscripts. A significantly small positive correlation between the number of references and the journal self-citations was noted (r(557) = 0.224; p < 0.001). On an international stage, the UK (0.63, 0.67 in JAMA Surg and ACS, respectively) and Israel (0.75 in AOS) had the highest average self-citation index across all three journals, with the UK repeating in both JAMA Surg and ACS; however, New Zealand (0), Canada (0), and Spain (0.11) had the lowest average self-citation indices across all three journals.

Conclusion: In the journals studied, both JAMA Surg and AOS were found to have equal levels of self-citation but significantly higher than ACS. Nearly a third of all the references across three journals were found to be first or last author self-citations. Additionally, journals self-cited at a lower rate, but a correlation existed between the number of references and journal's self-citation rate. A potential international co-localization of self-citations exists, especially among smaller European and Asian countries.

## Introduction

Citations are widely considered to be the backbone of a published paper and are an indicator of its significance, attention, and impact in the field [1]. In the past two decades, there has been a rising concern about the extent of author self-citation in correlation with research productivity performance indicators. The appearance of self-citations has long attracted bibliometric investigators, sociologists of science, and scientists [1]. Self-citation functions identically to other cited references appended to a paper; it credits publications on which the present work depends [1]. However, citations serve a dual function of receiving peer recognition and representing a symbol for specific original achievement [2]. Nevertheless, the act of self-citation remains uncertain as to whether it should be considered a legitimate source of information or if it should be discounted on the underlying premise of sui generis [1].

Although citations may reflect a genuine individual’s mark of influence, recent data have suggested that there is an increased misrepresentation of research performance by authors who self-cite to artificially inflate their Hirsch Index (h-index), a metric to quantify an author’s research impact based on the total number of publications and citations [1]. Similarly, the impact factor of a journal can be artificially inflated through journal self-citations, contributing to possible higher visibility and impact of the journal [3]. During the peer review process, editors may incentivize authors to cite from within the publishing journal or self-cite to increase the chances of manuscript acceptance [3]. This situation gives rise to apprehension regarding the growing presence of coercion within the scientific community, which in turn may exert influence on the author’s decision-making process regarding the inclusion of requested citations or failure to comply and potentially risk rejection of submission [4].

A multitude of excessive self-citation and “citation cartels” have been documented with numerous artificial forms of self-citation, such as an inflated h-index, impact factor, and even strategic planning among researchers from specific nations to significantly boost their metrics [4-8]. However, to the authors' knowledge, no study has been conducted on author or journal self-citation rates for physician-scientists of high-ranking journals in general surgery. This study aims to uncover the differences in author and journal self-citation rates in three high-impact general surgery journals for a time duration of one year.

## Materials and methods

Protocol

The university institutional review board deemed this research as not qualifying for human subjects research and approved the study protocol. A retrospective review of all original research articles from the Journal of the American Medical Association in Surgery (JAMA Surg), Annals of Surgery (AOS), and American College of Surgeons (ACS) published on January 1, 2022, to December 31, 2022, was manually collected following a structured abstraction method. Original research was defined as any article published from January 1, 2022, to December 31, 2022, under "Original Investigations" for JAMA Surg, "Original Articles" for AOS, and "Original Scientific Articles" in ACS. This excluded randomized controlled trials, featured articles, editorials, meta-analyses, review articles, or clinical reports. The following factors contributed to the three journals being chosen: the impact factor the journal held in 2022, relevance to general surgery as testified by an expert in the field, public availability of the articles within a journal, and publication in the English language.

Research assistants were trained for one week by the principal investigator with practice articles before initiating this study. All data were collected by three trained research assistants who recorded the total number of authors, references, author self-citations, journal self-citations, and nationality of the authors within each research article for the journal they were assigned, either JAMA Surg, AOS, or ACS. A self-citation is recorded if the research assistant can find the author's last name in the reference section. The same process was applied for journal self-citations. The complete author list was utilized to ensure that each citation was accounted for during data collection. Subsequently, every article was scrutinized by three different research assistants who were not involved with this study for the accuracy of the data collected, and every error encountered was corrected before any statistical analysis. 

Author self-citation was defined as an author of the original research article citing themselves in the reference section of that same article. The author self-citation rate was defined as the number of authors self-citing/the total number of authors [9]. A journal self-citation was recorded when the name of the journal within which the article was published was found in the reference list of the original research article [9]. The journal self-citation rate was defined as the number of times the specific journal was recorded in the references/the total number of references [9].

Statistical analysis

IBM SPSS Statistics for Windows, version 27.0 (IBM Corp., Armonk, NY) was used to analyze data, with statistical significance being defined as p ≤ 0.05. Results are reported descriptively with point estimates and a measure of distribution. The Kruskal-Wallis test, followed by a Mann-Whitney U, was used to assess continuous data between journals. Spearman’s correlation was used to evaluate the correlations between continuous variables, where r = 0.1 to 0.3, 0.3 to 0.5, and 0.5 to 1.0 are weak, moderate, and strong correlations, respectively.

The author’s nationality was analyzed utilizing a visual plotting of the data on a world map, formally called Geo Mapping. Each country's relative proportion of self-citation was defined as the number of authors who self-cite within a country/total number of authors within a country, where the denominator is determined by counting the number of authors from each country in our samples. This is labeled as the “average self-cite index” on a scale of 0 to 1. The same principle is specifically applied to the Geo Map average self-citation index for each state in the US for all journals.

## Results

In total, 558 original research manuscripts (JAMA Surg 107, AOS 322, ACS 129) were reviewed from the three journals in 2022, where the number in parentheses next to each journal represents the median value per journal. The total number of authors reviewed was 6,399, with a statistically significant difference noted for the number of authors per article between JAMA Surg and ACS (p = 0.001), which may skew results toward certain journals over others (Table 1). Post-hoc comparisons with the Mann-Whitney test revealed that articles published by ACS were published with a smaller author list than JAMA Surg (p = 0.01). In the entire cohort, there was a total of 28.4% (N = 1819) authors who self-cited at least once in the references of their research, with statistically significant difference between JAMA Surg and AOS vs. ACS, and no differences between JAMA Surg and AOS (JAMA Surg med = 3 and AOS med = 3 vs. ACS med = 2; p < 0.001) (Table 2). A significant positive correlation was noted between the total number of authors on an article and the number of authors who self-cite (r(556) = 0.386; p < 0.001). The total number of times authors self-cited was 3,852, with a statistically significant difference noted between journals (JAMA Surg med = 6 and AOS med = 6 vs. ACS med = 3; p < 0.001) (Table 1). The assessment of the number of self-citations by each journal showed JAMA Surg to have similar rates of self-citations compared to AOS, but both JAMA Surg and AOS had higher rates of self-citation than ACS (p < 0.001).

**Table 1:** Cumulative representation of author and journal self-citations rates for each journal Data are presented as the total sample and the median. JAMA Surg: JAMA Surgery; AOS: Annals of Surgery; ACS: Journal of the American College of Surgeons

	JAMA Surg	AOS	ACS	P-values
Articles	107	322	129	N/A
Number of authors	1346 (10)	3630 (9)	1423 (8)	p = 0.024
Number of authors who self-cite	390 (3)	1139 (3)	290 (2)	p < 0.001
Number of times authors self-cite	842 (6)	2468 (6)	542 (3)	p < 0.001
Number of times the first author self-cites	138(0)	444 (1)	129 (0)	p = 0.009
Number of times the last author self-cites	186 (1)	452 (1)	117 (0)	p < 0.001
Number of references	3715 (33)	11915 (35)	4313 (31)	p = 0.038
Number of times a journal self-cites	127 (0)	1056 (2)	154 (1)	p < 0.001

**Table 2:** Author and journal self-citation rates presented as percentages Data represented as percentage with calculation within each parenthesis. JAMA Surg: JAMA Surgery; AOS: Annals of Surgery; ACS: Journal of the American College of Surgeons

	Total	JAMA Surg	AOS	ACS
Author self-citation rate	28.4% (1819/6399)	28.9% (390/1346)	31.4% (1139/3630)	20.4% (290/1423)
Journal self-citation rate	6.7% (1337/19943)	3.4% (127/3715)	8.9% (1056/11915)	3.6% (154/4313)

The number of times a first author self-cited was 17.1% (N = 711), and the number of times a last author self-cited was 19.6% (N = 755) from the total number of times authors self-cited (p = 0.020 and p < 0.001, respectively) (Table 1). The first author's self-citation was most seen in AOS (med = 1), followed by JAMA Surg (med = 0.5) and then ACS (med = 0), with specific differences being noted between AOS and ACS (p = 0.005), represented by median values in parentheses. Last author self-citations were most common in JAMA Surg (med = 1) and AOS (med =1), followed by ACS (med = 0), with significant differences between JAMA Surg and ACS (p < 0.001) and AOS and ACS (p < 0.001) (Table 1). There was a significant, strong positive correlation between the number of times authors self-cited versus the number of times the first author self-cited (r(556) = 0.582, p < 0.001). In addition, there was a significant, strong positive correlation between the number of times authors self-cited versus the number of times the last author self-cited (r(556) = 0.634, p < 0.001).

The total number of references in all original manuscripts studied was 19,943, with a statistically significant difference noted between the journals (AOS med = 35 vs. ACS med = 31; p = 0.019) (Table 1). Journal self-citations accounted for 6.7% (N = 1,337) of all references, with significant differences between JAMA Surg (med = 0) and AOS (med = 2) (p < 0.001) and between AOS and ACS (med = 1) (p < 0.001) (Table 2). There was a significant, small positive correlation between the number of references and the journal self-citations (r(557) = 0.224; p < 0.001). 

**Figure 1:**
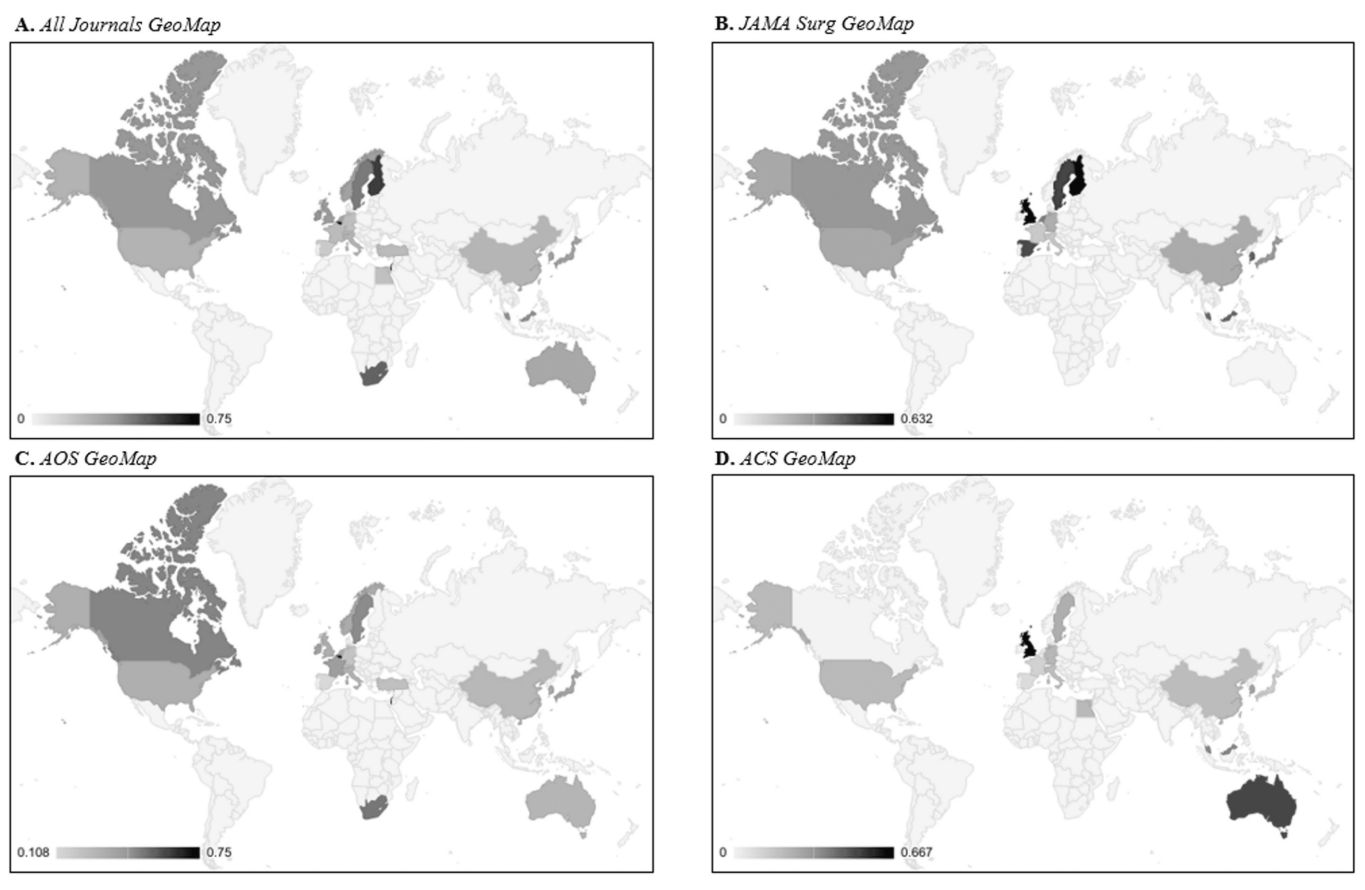
Mapping of the average self-citation index for every country represented in each journal 1A) Average self-citation index calculated across all journals representing all countries. 1B) Average self-citation index for countries within JAMA Surgery. 1C) Average self-citation index for countries within AOS. 1D) Average self-citation index for countries within J Am Coll Surg. JAMA Surg: JAMA Surgery; AOS: Annals of Surgery; ACS: Journal of the American College of Surgeons

The average self-cite index was utilized to map the relative contribution of each country toward their respective self-citation levels, represented on a color gradient: a lighter color represents low levels of average self-citation, while a darker color represents higher levels of average self-citation. The UK (0.63, 0.67 in JAMA Surg and ACS, respectively) and Israel (0.75 in AOS) had the highest average self-citation index across all three journals, with the UK repeating in both JAMA Surg and ACS (Figure 1A-1D). By contrast, New Zealand (0), Canada (0), and Spain (0.11) had the lowest average self-citation index for JAMA Surg, AOS, and ACS, respectively (Figure 1A-1D). Averaging the data across all three journals, Israel had the highest index while New Zealand retained the lowest (Figure 1A). 

**Figure 2:**
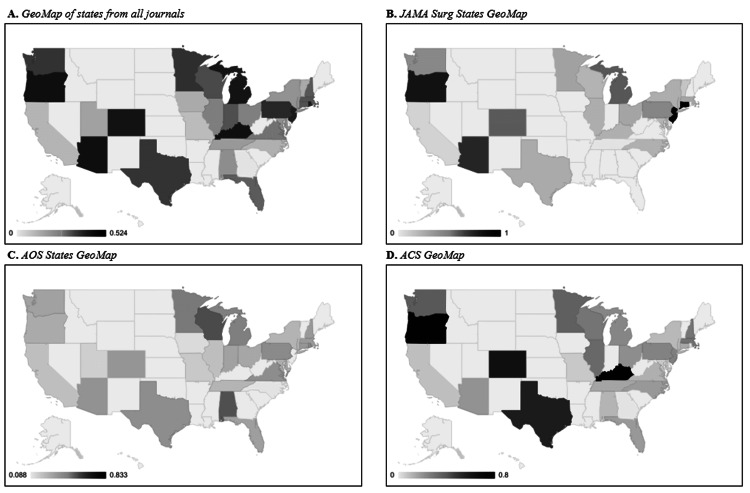
Mapping of average self-citation index for every state represented in each journal 2A) Average self-citation index calculated across all journals representing all states. 2B) Average self-citation index for states within JAMA Surgery. 2C) Average self-citation index for states within AOS. 2D) Average self-citation index for states within J Am Coll Surg. JAMA Surg: JAMA Surgery; AOS: Annals of Surgery; ACS: Journal of the American College of Surgeons

At a national level, a Geo Map was created for each journal to display the average self-citation for every state and territory in the US. There were differences noted in the index for states and territories across all three journals: Connecticut and New Jersey (1) from JAMA Surg, Washington D.C. (0.83) from AOS, and Kentucky (0.8) from ACS held the highest average self-cite index (Figure 2B-2D). This contrasted with Tennessee (0), North Carolina (0.09), and Georgia (0), having the lowest average self-cite index from JAMA Surg, AOS, and ACS, respectively (Figure 2B-2D). Combining the data across all three journals, Washington, D.C., had the highest index (0.53), and Georgia (0) retained the lowest index (Figure 2A).

## Discussion

Self-citation is generally accepted within the realm of narrow research topics, transfer of knowledge, and expansion of previous hypotheses, and it is allowable to have a median self-citation rate of 10-20% [10]. However, our current study found higher author and journal self-citation rates by authors who publish in JAMA Surg and AOS. The higher number of self-citing authors displayed in JAMA Surg and AOS than in ACS could be attributed to a multitude of reasons, such as the current manuscript being on a continuum of previous relevant publications, reviewers recommending legitimate, important research that may improve the author’s article, or potentially adding citations for self-promotion [11].

Author self-citation, both appropriate and inappropriate, has been practiced in medicine and other disciplines for a long time [12-14]. Early career scientists tended to have higher self-citation rates due to insufficient time to collect citations from other researchers, which is described as the “Youth Effect” [10]. With research output being an essential aspect of a surgeon’s career, it may pressure them to publish and be accepted for publication [15]. In addition to research pressures, many projects have a very narrow topic, requiring authors to self-cite to transfer knowledge [16]. In lieu of appropriate self-citations, inappropriate author self-citations to increase professional credibility [17-18] may lead to falsifying the importance and relevance of self-cited articles. 

The authors found a significant moderate positive correlation between the total number of authors' self-citations and the number of authors per manuscript in all three journals. This supports bibliometric studies done in cardiovascular medicine, where self-citations peaked for studies with more authors and smaller sample sizes [13]. The current study also uncovered a significant positive relationship between the total number of times authors self-cited versus the number of times the first and last authors self-cited in an article. It expounds upon the “Youth Effect” evidence where younger authors are more willing to self-cite to increase their h-index and amass more citations, which could lead to more credibility and potential support for funding in future research endeavors. The additional strong relationship with the last author's self-citation is crucial as it points toward the idea that the last author tends to have more experience in the specific research topic and more articles published that are like the topic. As a result, authors are more likely to cite the last author due to previous research existing for that author. 

The evaluations of these self-citations should also consider the potential for manipulating the h-index for self-promotion and increasing the chances of receiving more funding. This will misrepresent the author’s h-index and falsely improve their research influence. Although this is possible for any author and journal, evaluating the full scope of the h-index and intentions of self-citation efficiently is nearly impossible. To combat this issue, a self-citation index (s-index) that reports the self-citation rate per article along with the h-index for each paper could be used separately to represent self-citations [19]. Additionally, the ratio of citations received per paper to the number of papers with those citations can also be calculated [10]. This, in combination with the s- and h-index, will provide a method of more accurately uncovering potential excessive self-promotion [10]. 

In addition to author self-citations, there are significant differences in journal self-citation rates between JAMA Surg, AOS, and ACS. Table 1 shows that AOS has the highest median journal self-citation rate compared to both JAMA Surg and ACS, which concurs with prior findings that the proportion of papers citing from within the journal has been rising since 2004 [3]. However, it has also been reported that the journal’s impact factor has become more dependent on the number of citations it receives rather than solely relying on journal self-citations [3]. Although AOS shows greater journal self-citations, there are legitimate reasons why they may have a higher journal self-citation rate. First, a reader may find a specific journal topic interesting enough to decide to conduct another research study based on that topic. As a result, they are more likely to publish the article within the same journal from which they got inspiration. Second, the reader will add those same articles to their reference list since they know that the journal has a recent interest in that topic, increasing the overall journal self-citation rate [20].

In addition, it has been found that higher-ranked journals seemed more willing to take the risk to encourage potential authors into journal self-cites compared to lower-ranked journals [4]. High-impact factors aid a journal and can be used as a marketing tool, allowing for its articles to gain more popularity amongst its readers. More studies need to be done on a longer time scale and a wider variety of journals covering both high and low-impact factors to flesh out these nuances. This would provide the opportunity for further research in evaluating whether male or female authors are more likely to self-cite, which may be correlated with differences in promotion rates at academic centers.

In conjunction with higher author self-cite rates, a geographical pattern is associated with JAMA Surg, AOS, and ACS self-citation rates. On the international scale, numerous European countries, South Korea, Japan, Singapore, and Oceania showed greater average self-citation than the US, Canada, and China. There is a preference for scientists to collaborate with peers in their geographic areas, with citation and collaboration being strongly correlated [21]. Higher levels of cooperation between two cities lead to a proportional increase in the exchange of citations between the cities and, subsequently, a natural bias for self-citation [21]. We can extrapolate further that since smaller countries, such as European or Asian countries, have smaller areas by land, they will have a greater likelihood of collaborating between the same cities and more self-citations [21]. These countries also have lower research output, so their average self-citation index increases mathematically, as evidenced by the smaller countries in Figure 1 having a low number of original articles but high levels of self-citation. 

Due to the three journals being primarily housed in the US, mapping the self-citation by states gave a better understanding of regionality preferences for self-citation within our own country. From a broad perspective, all three maps showed a much larger cluster of states on the east coast with higher self-citation indices than the west coast states. More specifically, east coast states such as Pennsylvania, New Jersey, Rhode Island, Michigan, Florida, and many others tended to have higher average self-citation indices compared to a few west coast states such as Arizona, Oregon, and Washington State. Like the previously discussed countries, smaller states may show an increased flow of citations between collaborators in contrast to the larger western coast states. 

Limitations

This study has important limitations. One of the limitations of the current analysis is the need for adequate original articles to draw a significant conclusion regarding the geographical patterns we see. Ideally, the authors would also want to increase the length of time during which data were collected and expand to other journals to get an enhanced view of regionality preferences for self-citations. Likewise, only original articles from three general surgery journals within a one-year period were studied. The findings of this study may not be extrapolated to other article types or other general surgery journals. There may also be inaccuracies in the self-citation number for authors with common names. The sheer amount of data collected from journals prevents the accurate evaluation of an author’s work from checking for appropriate self-citations, as their work was limited to the inclusion criteria of original research manuscripts. If other manuscript types had been included in this review, the author and journal self-citation rates might have differed. 

## Conclusions

This study elucidates notable disparities in author and journal self-citation practices among three high-impact general surgery journals, i.e., JAMA Surg, AOS, and ACS. Author self-citation rates were significantly higher in JAMA Surg and AOS, a pattern influenced by manuscript authorship structure, particularly the involvement of first and last authors. The observed correlations suggest that increasing author count amplifies opportunities for self-citation, reflecting both collaborative dynamics and the influence of established researchers. Journal self-citation patterns also varied meaningfully, with AOS exhibiting the highest rates. Geographic analyses revealed distinct self-citation behaviors, with smaller nations and several US. East Coast states demonstrating elevated average self-citation indices - likely reflecting regionally concentrated research activity and tighter institutional networks.

Together, these findings underscore the multifaceted nature of self-citation in academic surgery. While often a legitimate expression of intellectual continuity, self-citation also operates within a broader ecosystem shaped by scholarly recognition, institutional expectations, and bibliometric valuation. Understanding these dynamics is essential for interpreting citation-based metrics within the context of academic influence and research integrity.
